# Kidney Perfusion in Contrast-Enhanced Ultrasound (CEUS) Correlates with Renal Function in Living Kidney Donors

**DOI:** 10.3390/jcm11030791

**Published:** 2022-02-01

**Authors:** Nasrin El-Bandar, Markus H. Lerchbaumer, Robert Peters, Andreas Maxeiner, Katja Kotsch, Arne Sattler, Kurt Miller, Thorsten Schlomm, Bernd Hamm, Klemens Budde, Lutz Liefeldt, Thomas Fischer, Frank Friedersdorff

**Affiliations:** 1 Department of Urology, Charité–Universitätsmedizin Berlin, Corporate Member of Freie Universität Berlin, Humboldt-Universität zu Berlin and Berlin Institute of Health, 10117 Berlin, Germany; nasrin.el-bandar@charite.de (N.E.-B.); robert.peters@charite.de (R.P.); andreas.maxeiner@charite.de (A.M.); kurt.miller@charite.de (K.M.); thorsten.schlomm@charite.de (T.S.); 2 Department of Radiology, Charité–Universitätsmedizin Berlin, Corporate Member of Freie Universität Berlin, Humboldt-Universität zu Berlin and Berlin Institute of Health, 10117 Berlin, Germany; markus.lerchbaumer@charite.de (M.H.L.); bernd.hamm@charite.de (B.H.); thom.fischer@charite.de (T.F.); 3 Department of General, Visceral and Vascular Surgery, Charité–Universitätsmedizin Berlin, Corporate Member of Freie Universität Berlin, Humboldt-Universität zu Berlin and Berlin Institute of Health, 10117 Berlin, Germany; katja.kotsch@charite.de (K.K.); arne.sattler@charite.de (A.S.); 4 Department of Nephrology and Internal Intensive Care Medicine, Charité–Universitätsmedizin Berlin, Corporate Member of Freie Universität Berlin, Humboldt-Universität zu Berlin and Berlin Institute of Health, 10117 Berlin, Germany; klemens.budde@charite.de (K.B.); lutz.liefeldt@charite.de (L.L.); 5 Department of Urology, Evangelisches Krankenhaus Königin Elisabeth Herzberge, 10365 Berlin, Germany

**Keywords:** Contrast-enhanced ultrasound, kidney perfusion, kidney function, kidney transplantation, kidney donation

## Abstract

Contrast-enhanced ultrasound (CEUS) is a widely used diagnostic tool for analyzing perfusion and characterizing lesions in several organs. However, to date, it has not been sufficiently investigated whether there is an association between CEUS findings and kidney function. This study aimed at identifying the potential relationship between kidney function and the renal perfusion status determined by CEUS in living kidney donors. A total of 30 living kidney donors examined between April 2018 and March 2020 were included in the study. All patients underwent various diagnostic procedures for evaluation of renal function. CEUS was performed in all 30 donors one day before nephrectomy. Kidney perfusion was quantified using a postprocessing tool (VueBox, Bracco Imaging). Various perfusion parameters were subsequently analyzed and compared with the results of the other methods used to evaluate kidney function. Of all parameters, mean signal intensity (MeanLin) had the strongest correlation, showing significant correlations with eGFR (CG) (r = −0.345; *p* = 0.007) and total kidney volume (r = −0.409; *p* = 0.001). While there was no significant correlation between any perfusion parameter and diethylenetriaminepentaacetic acid (DTPA), we detected a significant correlation between MeanLin and DTPA (r = −0.502; *p* = 0.005) in the subgroup of normal-weight donors. The results indicate that signal intensity in CEUS is associated with kidney function in normal-weight individuals. Body mass index (BMI) may be a potential confounder of signal intensity in CEUS. Thus, more research is needed to confirm these results in larger study populations.

## 1. Introduction

Evaluation of kidney function is crucial in living donor candidates, who should not be exposed to avoidable health impairments. The Amsterdam Guidelines recommend a glomerular filtration rate (GFR) ≥80 as an essential prerequisite for living kidney donation [1]. Accurate assessment and verification of adequate kidney function is essential during evaluation of a potential kidney donor [1]. Several methods are currently in clinical use for assessment of kidney function with differences in accuracy, complexity and duration [2]. However, accurate assessment of kidney function is not only needed in the context of living kidney donation. More importantly, it is needed whenever impairment of kidney function is suspected.

In routine clinical practice, the most frequently used method is measurement of serum creatinine. Determination of serum creatinine for assessment of renal function is limited by the fact that an increase only becomes evident after 50 % of kidney function is lost [2]. Estimated glomerular filtration rate (eGFR) is also widely used and provides a more accurate assessment of kidney function using one of the established formulas, such as “modification of diet in renal disease“ (MDRD), “Cockcroft–Gault“ (CG) or the “chronic kidney disease epidemiology collaboration equation“ (CKD-EPI) [3,4,5,6]. Especially in the context of living kidney donation, use of an estimation formula for assessing kidney function is widely considered as imprecise and not appropriate [1,7,8,9,10]. Radioisotopic techniques provide more accurate information on kidney function and are widely used to measure split and total renal function [10,11]. Radiotracers that are used as exogenous filtration markers to measure GFR include ^51^Cr-ethylenediaminetetraacetic acid (EDTA), ^99m^Tc-diethylenetriaminepentaacetic acid (DTPA), ^99m^Tc-mercaptoacetyltriglycine (MAG3) and ^125^I-iothalamate [2,10].

Besides the widely used and established methods for the evaluation of kidney function, various imaging techniques have been reported to significantly correlate with kidney function—including measurement of cortical volume or total kidney volume on computed tomography (CT), measurement of kidney length in ultrasound (US) and assessment of kidney function in dynamic contrast-enhanced magnetic resonance imaging (MRI) [10,12,13,14]. However, it has not been sufficiently investigated if there is a relationship between kidney perfusion in contrast-enhanced US (CEUS) and kidney function. Since hyperperfusion can be considered an early sign of glomerular injury, kidney perfusion in CEUS could possibly provide information for detection of early kidney injury—especially when kidney function appears to be normal [15,16,17]. Thus, a proportion of the patients may be spared an invasive procedure for histological confirmation of kidney injury.

To date, CEUS has been primarily performed to characterize focal renal lesions and assess perfusion patterns based on microvascularization following administration of a strictly intravascular contrast agent [18,19]. Since CEUS has not yet been sufficiently investigated in the context of whole-organ perfusion and kidney function, this pilot study was conducted to analyze the potential relationship between kidney function and CEUS in living kidney donors.

## 2. Material and Methods

### 2.1. Study Design and Patient Population

The study was designed as a feasibility study to investigate a potential relationship between kidney function and kidney perfusion in CEUS. For this reason, 30 living kidney donors underwent CEUS one day prior to donor nephrectomy. Overall, 60 kidneys were examined between April 2018 and March 2020. The study was approved by the local institutional review board (Ethical Committee of Charité Universitätsmedizin Berlin) (EA1/406/16) and conformed to the amended Declaration of Helsinki. All donors were informed about the procedure and possible risks 24 h before the examination and provided written informed consent. The results of this study were not used to guide clinical management.

All living kidney donor candidates invariably undergo evaluation of total and split renal function using radioisotopic techniques prior to nephrectomy in our hospital. DTPA clearance was determined for assessment of total kidney function and MAG3-scintigraphy was used for evaluation of split renal function. Additional examination of kidney perfusion by CEUS enabled a direct comparison between CEUS-derived perfusion parameters and various established methods for evaluation of kidney function in individuals with healthy kidneys.

### 2.2. CEUS Examination Protocol

All examinations were performed by three examiners with many years of experience in CEUS, together with two assistants who administered the ultrasound contrast agent (UCA). SonoVue^®^ (Bracco Imaging, Milan, Italy) was used as a second-generation UCA for all examinations and was administered via a 3-way stopcock in the antecubital vein, followed by a saline flush. The UCA was prepared according to the manufacturer’s instructions.

**Figure 1:**
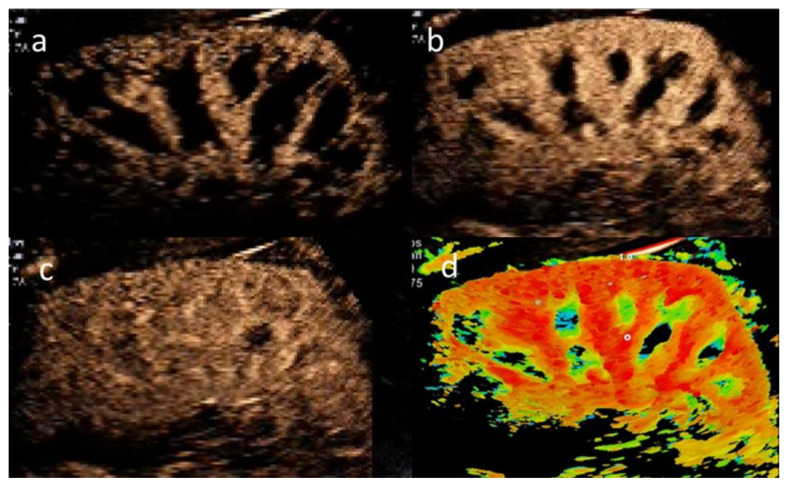
Example illustrating kidney perfusion in CEUS approximately 1 s (**a**), 4 s (**b**) and 13 s (**c**) after detection of the first signal (approximately 10–15 s after injection of the ultrasound contrast agent). In (**a**) the contrast agent enhances the interlobar arteries and part of the renal cortex. Full cortical enhancement is seen in (**b**), and perfusion of the whole kidney including the renal pyramids is shown in (**c**). Figure (**d**) shows the time course of successive enhancement in different colors in a single image. Red indicates earliest enhancement, followed by yellow, green and blue regions.

All CEUS examinations were performed using a high-end ultrasound system (Aplio i900, Canon, Otawara, Japan) with a contrast-specific mode and the same multifrequency (i8CX1). CEUS was performed of the right kidney, followed by the left kidney. After positioning the transducer for renal imaging in the longitudinal plane in deep inspiration, a 1.6 mL UCA bolus was injected, followed by a rapid 10 mL saline flush. Approximately 10 to 20 s (s) after UCA administration, the first microbubbles appeared in the interlobar arteries, followed by rapid filling of the renal cortex and prolonged medullar enhancement (Figure 1). After the first contrast signal was displayed, microflow kinetics were recorded as a 30-s loop during a single breath hold. Since pulmonary elimination of the applied UCA takes approximately 5 to 10 min, CEUS of the left kidney was performed after a 10-min waiting time and when no CEUS signal was apparent in the left kidney.

### 2.3. Ultrasound Settings

The UCA consists of microbubbles [20]. To avoid microbubble destruction, the UCA was administered in a straight direction. Moreover, scanning was performed with a low mechanical index (MI; 0.07–0.09) [21]. Beside the MI, there are other settings that may have influenced the received signal intensity. In this study population, Gain (G) ranged between 76 and 89 and the dynamic range (DR) was either 75 or 60.

### 2.4. Quantitative Perfusion Analysis

CEUS cine loops were stored as DICOM raw data and transferred to a software package for further analysis. Quantitative analysis of kidney perfusion with time-intensity curve measurements (TIC) was performed using the VueBox^®^ postprocessing software package (Bracco Imaging, Milan, Italy). Motion compensation—which is offered by the software as an option for optimization of quality of fit (QOF)—was applied to all videos analyzed. Except for three video clips analyzed with QOF of 88, 78 and 74, QOF was over 90.

After motion compensation, a freehand region of interest (ROI) was manually placed in the renal cortex by the same person excluding artifacts. To ensure optimal comparability, the positions of the drawn ROIs had to be consistent. Therefore, all ROIs were drawn in a central position (middle third of the kidney) with adequate image quality. Based on the ROIs, the software generated time-intensity curves and computed different perfusion parameters.

### 2.5. Perfusion Parameters

**Figure 2:**
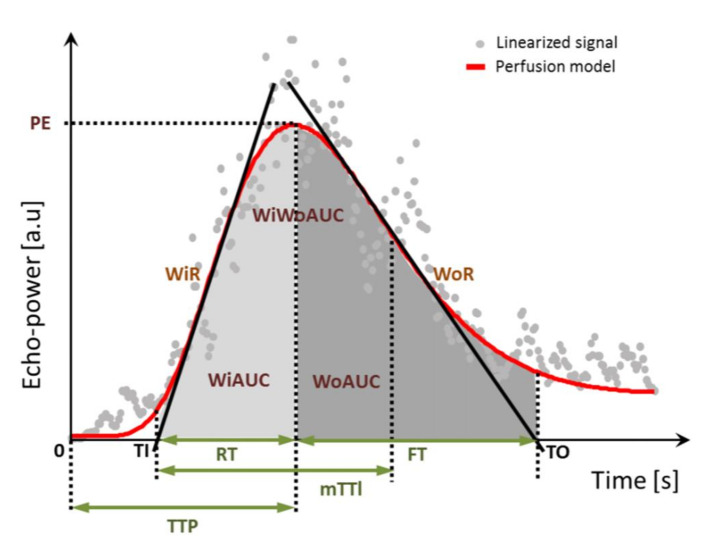
CEUS perfusion parameters in a model. TI defines the point in time when the tangent of the maximum increase (WiR) intersects the x-axis. TO is the point in time when the tangent of the maximum decrease intersects the x-axis. Parameters shown are described in Table 1 and Table 2. WiWoAUC: Wash-in and wash-out Area Under the Curve; WoR: Wash-out Rate; WiR: Wash-in Rate; WoAUC: Wash-out Area Under the Curve; WiAUC: Wash-in Area Under the Curve; PE: Peak Enhancement; RT: Rise time; FT: Fall time; mTT1: Mean transit time local (mTT-Tl); TTP: Time to peak; S: seconds; a.u: arbitrary units; Figure source: VueBox—instruction for use [19]. CEUS: Contrast-enhanced ultrasound.

Figure 2 shows the perfusion parameters automatically determined by VueBox^®^ (Bracco Imaging, Milan, Italy). [22]. They can be classified into two categories: signal intensity parameters and time-related parameters (Table 1). Signal intensity parameters, such as peak enhancement (PE) and area under the curve (AUC), are determined to describe relative blood volume and mean transit time (mTTl) as a time-related parameter describes the mean blood flow velocity [21]. For simplicity’s sake, and since signal intensities were very high, signal intensity parameters were divided by 1000.

### 2.6. Patient Data and Methods for Assessment of Kidney Function

Different methods for evaluation of kidney function were applied and the results documented for comparison with CEUS-derived perfusion parameters. MAG3-scintigraphy, DTPA clearance and serum creatinine were collected from the hospital’s general documentation system. eGFR was calculated according to the CG, MDRD and CKD-EPI formulas [3,4,5]. Split kidney volume was determined by an automatic calculation after manually framing the kidneys in CT in all representative slices. Total kidney volume was calculated by adding both right and left split kidney volume. BMI was used to categorize donors into normal weight (18.5–24.9 kg/m^2^), overweight (25–29.9 kg/m^2^) and obesity (≥30 kg/m^2^). A total of 15 patients belonged to the normal-weight group.

DTPA clearance was used as the reference standard for measurement of kidney function. Donors were instructed to drink at least one liter prior to DTPA clearance testing. DTPA clearance was determined using the Fleming formula [23]. MAG3 scintigraphy was performed to determine the proportion of split renal function for each kidney based on tubular extraction rate (TER) according to Bubeck [24]. Split DTPA clearance was calculated by multiplying the proportion of split kidney function derived from MAG3-scintigraphy with absolute DTPA clearance. Split eGFR was also calculated by multiplication with the result of MAG3-scintigraphy.

### 2.7. Statistical Analysis

First, established methods for evaluation of total kidney function prior to nephrectomy were compared with each other as a basis for comparing the results of these methods with CEUS-derived parameters and determining the degree of correlation. We analyzed whether CEUS parameters were related to (1) total kidney function prior to nephrectomy, (2) split kidney function prior to nephrectomy and (3) total kidney function after nephrectomy. For analysis of a potential relationship between CEUS parameters and postoperative total kidney function, CEUS parameters derived from the retained kidney before donor nephrectomy were used. Finally, confounders that could have an influence on CEUS parameters were analyzed. To investigate potential relationships between kidney function and renal perfusion in CEUS, Pearson correlations were carried out. Descriptive results are presented as mean +/− standard deviation.

A two-sided significance level of α = 0.05 was defined to indicate statistical significance. All statistical analyses were performed using the SPSS software package (IBM Corp. Released 2016. IBM SPSS Statistics for Windows, Version 26.0. Armonk, NY, USA: IBM Corp.).

## 3. Results

### 3.1. Epidemiology and Descriptive Presentation of Kidney Function Data

Donors had a mean age of 54 +/− 9 years with a range of 37–75. The majority were female (76.7%), and mean BMI in the study population was 26 kg/m^2^ (+/−4 kg/m^2^).

Table 2 provides an overview of the kidney function tests performed both before and after nephrectomy. Prior to nephrectomy, mean DTPA clearance was 97 mL/min/1.73 m^2^ (+/−14). Estimation of GFR yielded lower values than DTPA clearance. Among all estimation formulas, the CG formula yielded the mean value closest to that obtained by determination of DTPA clearance; however, scatter was also greatest with a standard deviation of 25 mL/min/1.73 m^2^.

### 3.2. CEUS Parameters

Table 3 compiles the CEUS-derived values assessing signal intensity and time. Overall, there was large scatter of signal intensity parameters. All signal intensity parameters correlated strongly and significantly with MeanLin as a representative signal intensity parameter (r = 0.930 to r = 0.984; *p* < 0.001). In addition, CEUS-derived, time-related parameters also showed strong and significant correlation with each other. For correlation of time-related parameters, rise time (RT)—as a marker of arterial inflow—was chosen as a representative parameter.

### 3.3. Analysis of Total Kidney Function

Established methods for evaluation of total kidney function prior to nephrectomy were compared with each other. Table 4 shows correlations of various kidney function tests with DTPA clearance as the reference method for evaluation of total kidney function. DTPA clearance showed the strongest and most significant correlation with eGFR (CG) (r = 0.531; *p* = 0.003). In comparison, just a weak correlation was observed for serum creatinine and DTPA clearance (r = −0.228; *p* = 0.225). Because eGFR (MDRD) and serum creatinine showed only weak correlations, these parameters were not considered for further analysis.

### 3.4. Comparison of CEUS and Kidney Function Parameters

Table 5 compiles correlations of CEUS signal intensity parameters with the results of different kidney function tests. All signal intensity parameters showed weak and nonsignificant correlations with DTPA and eGFR (CKD-EPI). Conversely, all signal intensity parameters correlated significantly with eGFR (CG) and total kidney volume. MeanLin (r = −0.345; *p* = 0.007 and r = −0.409; *p* = 0.001) and WiWoAUC (r = −0.346; *p* = 0.008 and r = −0.401; *p* = 0.002) showed the strongest correlations. In contrast to correlations between established methods for evaluation of total kidney function, correlations with CEUS signal intensity parameters were invariably negative. Higher signal intensities were associated with lower kidney function. These correlations were comparable with the correlation between DTPA clearance and eGFR (MDRD) and even stronger than the correlation between serum creatinine and DTPA clearance. Overall, correlations with total kidney volume were stronger than correlations with eGFR (CG). Time-related CEUS parameters did not show any significant correlation with kidney function test results (Table 5). Thus, these CEUS parameters were not taken into consideration for further analysis.

In the assessment of preoperative split kidney function, split kidney volume and split eGFR (CG) also correlated with signal intensity parameters in CEUS (Table 6). Similarly, postoperative kidney function—to be precise eGFR (CG)—significantly correlated with CEUS intensity parameters of the remaining kidney (Table 6). For both preoperative split kidney function and postoperative kidney function correlation values were close to those for total kidney function.

### 3.5. Evaluation of Confounders

**Figure 3:**
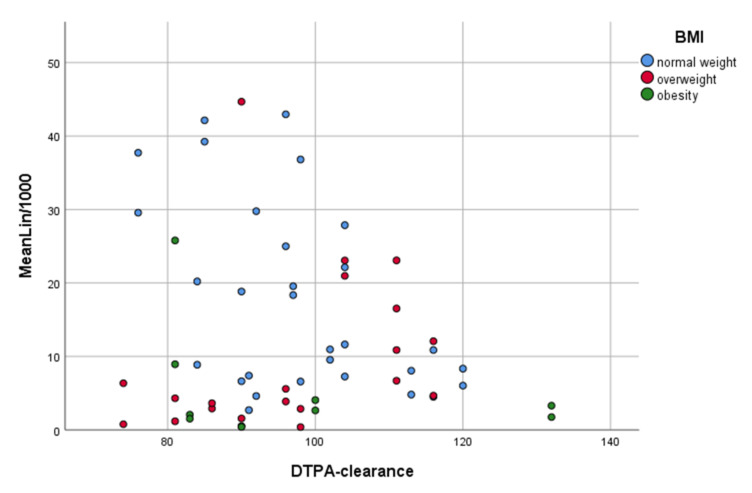
Scatter diagram of the association between MeanLin and DTPA clearance. Each dot represents one kidney. Data are presented in different colors for each of the three BMI-based subgroups. The results suggest that BMI could have had an impact on signal intensity and might thus be a confounder affecting the relationship between MeanLin and DTPA clearance.

Although CEUS parameters showed significant correlations with eGFR (CG) and kidney volume, no correlations with the reference method DTPA clearance were detected. For identification of potential confounders, relationships of MeanLin and DTPA were depicted in a scatter diagram using three different colors to represent the three BMI subgroups investigated (Figure 3). In this diagram, higher values for MeanLin were associated with lower values for DTPA. On the other hand, smaller values for MeanLin were related to both high and small values for DTPA. However, the diagram also reveals that BMI may have had an impact on signal intensity. Donors with a higher BMI had low values for MeanLin, and no relation between MeanLin and DTPA could be identified. However, for normal-weight donors, the diagram shows a relation between MeanLin and DTPA (i.e., kidney function). The scatter diagram suggests that, in the normal-weight subgroup, higher values for MeanLin were associated with poorer kidney function, and lower values for MeanLin were associated with better kidney function.

Further analysis showed a significant negative correlation between BMI and MeanLin (r = −0.366; *p* = 0.004). A larger BMI was therefore associated with smaller MeanLin values.

Patients with normal weight (n = 15, defined as BMI of 18.5–24.9 kg/m^2^) were selected to further analyze the relationship between DTPA and MeanLin. In this subgroup (30 kidney in total), MeanLin showed a significant and strong correlation with DTPA clearance (r = −0.502; *p* = 0.005).

## 4. Discussion

Although CEUS has been widely used in various clinical specialties, it has not been sufficiently investigated whether there is a relationship between CEUS-derived perfusion and kidney function. To our knowledge, this is the first standardized and prospective study to analyze the potential relationship between kidney function and CEUS-based perfusion parameters in individuals with healthy kidneys. Our study revealed significant correlations between different methods for evaluating kidney function and CEUS signal intensity parameters. However, no correlations were identified with time-related CEUS parameters. MeanLin was analyzed as a representative signal intensity parameter and showed the strongest correlations, which were similar for preoperative total kidney function, preoperative split kidney function and postoperative kidney function.

Significant correlations were only identified for total kidney volume and eGFR (CG), but not for the reference method—DTPA clearance. Because of the importance of the correlation between CEUS and the reference method for evaluation of kidney function, we analyzed potential confounders for their effects on the relation between DTPA clearance and signal intensity in CEUS.

Since greater penetration depth can attenuate the signal in CEUS, BMI was analyzed as a potential confounder [21]. Indeed, there was a strong correlation between MeanLin and DTPA clearance (r = −0.502; *p* = 0.005) in the normal-weight subgroup.

While MeanLin did not correlate significantly with DTPA clearance in the total study population, it showed significant correlation with eGFR (CG). The estimation formula used for calculation of eGFR (CG) is the only formula that incorporates information on body weight [5]. This may explain why MeanLin, as a parameter that can be influenced by BMI and thus by body weight, was found to significantly correlate with eGFR (CG).

There was a large scatter for all signal intensity parameters. BMI and kidney depth may have contributed to the large scatter. However, the failure to use consistent US system settings may also have contributed to differences in signal intensities. Mechanical index (MI), frames per second (fps), gain (G) and dynamic range (DR) varied between the CEUS examinations performed in our study population. The fact that ultrasound system settings, in general, affect signal intensity, and may both attenuate and enhance it, hampers comparison of absolute signal intensities [21,25].

Participation of three different examiners may also have contributed to the observed variability in signal intensities. Both inter- and intra-observer variability have been described for CEUS before [26]. However, the fact that CEUS was performed by different examiners alone cannot explain the large scatter.

Another potential confounder of signal intensity is the amount of fluid intake prior to a CEUS examination. In our hospital, patients scheduled for radioisotopic measurement are instructed to drink one liter of water before the examination since the amount of drinking may influence results. In contrast, no recommendation was made regarding fluid intake prior to the CEUS examination in this pilot study. As a result, fluid intake may have influenced signal intensities in CEUS.

According to our results, kidney volume may also influence signal intensities in CEUS. A large kidney appears to attenuate the CEUS signal. The underlying mechanism should be addressed in future studies.

To our knowledge, two studies have been published that investigated possible associations between renal function and perfusion in CEUS [17,27]. Both studies were conducted in patients with diabetic kidney disease (DKD) in comparison to control groups. Similar to our study, patient groups were small, with 33 and 55 patients with diabetic nephropathy. One of these studies, conducted by Ma et al., showed a positive correlation between GFR and the area under the curve (AUC) determined as a signal intensity parameter in CEUS [27]. Wang et al. also reported a significantly increased area under the ascending curve for patients with early-stage DKD (eGFR (MDRD) ≥90 mL/min/1.73 m^2^) compared to patients with moderate DKD (30–90 mL/min/1.73 m^2^) [17]. In contrast, our results showed a negative correlation between kidney function and CEUS-derived signal intensity parameters. However, we only investigated kidney function in individuals without underlying kidney disease, while the two earlier studies analyzed kidney function and CEUS-based perfusion in patients with DKD.

Future studies should consider comparison of absolute values with scintigraphic results as presented by Krumm et al. for contrast-enhanced MRI [27]. In general, ultrasound contrast agents are associated with very low adverse event rates and do not interact with renal function or lead to contrast-agent nephropathy [28]. Moreover, microbubble-based contrast agents do not interact with thyroid function as they do not contain iodine. Ultrasound contrast agents are proven as strictly intravascular, allowing the assessment of organ perfusion on microcirculation level. [21] This advantage, combined with a dynamic examination, settles a further argument for CEUS compared to CT and MRI, since iodinated and gadolinium-based contrast media are known to be not strictly intravascular. Nevertheless, CEUS and US are known to be operator-dependent, especially in image/cineloop acquisition for parametric measurements. Although CEUS and US, in general, possess advantages, such as the absence of radiation, lower costs and high availability (nearly every mid-range system contains CEUS specific software), both tomographic modalities (i.e., CT, MRI) are more standardized in the assessment of scans or sequences. Thus, a volume dataset and not a single plane alone (as in US) can be used for whole organ perfusion. Our results demonstrated the potential influence of BMI or body weight on the parametric assessment of renal perfusion in CEUS (driven by lower image quality at higher penetration depth). These confounders may not affect image quality and parametric evaluation of organ perfusion in tomographic imaging.

### Limitations

The most important limitation of this study is the small number of subjects included in this pilot study. Moreover, the lack of use of consistent US system settings and the involvement of several examiners could have affected our results. Additionally, since only patients in the subgroup of normal-weight donors showed a strong and significant correlation between MeanLin and DTPA clearance, a broad usage of CEUS is not applicable. Future studies are needed to analyze confounders and correlations in a larger population to enable a broad and standardized usage of this method.

## 5. Conclusions

This pilot study, for the first time, demonstrated a significant relationship between kidney perfusion in CEUS and kidney function in living kidney donors. However, its significance is limited to patients of normal weight and BMI, and the US settings appear to affect renal perfusion quantified by TIC measurements on CEUS. This pilot study provides important new insights and supports the role of CEUS in assessing whole organ perfusion. Future research should analyze the relationships and potential confounders identified here in a larger population.

## Figures and Tables

**Table 1:** CEUS parameters.

	CEUS Parameter	Label	Description
Time-related parameters †	RT	Rise time	-
	mTTl	Mean transit time local (mTT-Tl)	-
	TTP	Time to peak	-
	FT	Fall time	-
Signal intensity parameters *	MeanLin	-	Mean signal intensity
	PE	Peak enhancement	Maximum signal enhancement
	WiAUC	Wash-in area under the curve (AUC (TI:TTP))	AUC (area under the curve) during wash-in of UCA (between TI and TTP)
	WiR	Wash-in rate	Maximum increase
	WiPI	Wash-in perfusion index	WiAUC/RT
	WoAUC	Wash-out AUC (AUC (TTP:TO))	AUC (area under the curve) during wash-out of UCA (between TTP und TO)
	WiWoAUC	Wash-in and wash-out AUC	WiAUC + WiWoAUC
	WoR	Wash-out rate	Maximum decrease

* Signal intensity parameters in arbitrary units (a.u.). † Time-related parameters in seconds (s).

**Table 2:** Results for total and split kidney function.

			Mean	Standard Deviation	Total
Total kidney function prior to nephrectomy	DTPA clearance (mL/min/1.73 m^2^)		97	14	30
	Serum creatinine (mg/dL)		0.83	0.13	30
	eGFR (CG) (mL/min/1.73 m^2^)		95	25	30
	eGFR (CKD-EPI) (mL/min/1.73 m^2^)		87	14	30
	eGFR (MDRD) (mL/min/1.73 m^2^)		84	14	30
	Total kidney volume (cm^3^)		323	74	30
Split kidney function prior to nephrectomy	Proportion in MAG3 (%)	right	47.6	3.3	30
		left	52.4	3.3	30
	Split DTPA clearance (mL/min/1.73 m^2^)		49	8	60
	Split eGFR (CG) (mL/min/1.73 m^2^)		47	13	60
	Split eGFR (CKD-EPI) (mL/min/1.73 m^2^)		44	8	60
	Split eGFR (MDRD) (mL/min/1.73 m^2^)		42	8	60
	Split kidney volume (cm^3^)		162	38	60
Total kidney function after nephrectomy	eGFR (CG) (mL/min/1.73 m^2^)		59	17	30
	eGFR (CKD-EPI) (mL/min/1.73 m^2^)		50	11	30
	eGFR (MDRD) (mL/min/1.73 m^2^)		49	9	30

Calculation of split DTPA clearance and split eGFR by multiplication with the proportion of split kidney function in MAG3-scintigraphy; no depth correction was applied for calculation of split kidney function.

**Table 3:** Computed CEUS values.

Parameter *	Mean	Interquartile Range	Correlation with MeanLin	Total
MeanLin †	12.9	17.1 (3.7–20.8)	-	60
PE †	27.6	38.1 (8–46.1)	r = 0.984; *p* < 0.001	59
WiAUC †	80	102.1 (22.4–124.5)	r = 0.987; *p* < 0.001	59
WiR †	8.8	13.4 (2.3–15.7)	r = 0.937; *p* < 0.001	59
WiPI †	17.5	23.8 (5.6–29.4)	r = 0.985; *p* < 0.001	59
WoAUC †	150.8	192.3 (41.3–233.6)	r = 0.983; *p* < 0.001	58
WiWoAUC †	230.6	292 (64–356)	r = 0.983; *p* < 0.001	58
WoR †	3.7	5.3 (0.9–6.2)	r = 0.930; *p* < 0.001	58
	**Mean**	**Interquartile Range**	**Correlation with RT**	**Total**
RT ‡	4.7	1.2 (3.8–5)	-	59
mTTI ‡	25.8	17.1 (14–31.1)	r = 0.387; *p* = 0.002	59
TTP ‡	7.4	2.1 (6.2–8.3)	r = 0.860; *p* < 0.001	59
FT ‡	9.5	2.2 (7.5–9.7)	r = 0.898; *p* < 0.001	58

† values in (a.u.); ‡ values in (s). Since the actual values were very large, they were divided by 1000. * All abbreviations are explained in Table 1.

**Table 4:** Parameters used for evaluation of kidney function prior to donor nephrectomy.

	Correlation with DTPA Clearance	*p*-Value
eGFR (CG)	r = 0.531	*p* = 0.003
Total kidney volume	r = 0.472	*p* = 0.008
eGFR (CKD-EPI)	r = 0.470	*p* = 0.009
eGFR (MDRD)	r = 0.377	*p* = 0.040
Serum creatinine	r = −0.228	*p* = 0.225

**Table 5:** Correlation of CEUS parameters with different methods for evaluation of preoperative total kidney function.

	Correlation with DTPA	Correlation with eGFR (CKD-EPI)	Correlation with eGFR (CG)	Correlation with Total Kidney Volume
MeanLin	r = −0.170; *p* = 0.194	r = −0.179; *p* = 0.172	**r = −0.345**; ***p* = 0.007**	**r = −0.409**; ***p* = 0.001**
PE	r = −0.162; *p* = 0.221	r = −0.182; *p* = 0.169	r = −0.322; *p* = 0.013	r = −0.392; *p* = 0.002
WiAUC	r = −0.189; *p* = 0.152	r = −0.214; *p* = 0.104	r = −0.339; *p* = 0.009	r = −0.402; *p* = 0.002
WiR	r = −0.112; *p* = 0.399	r = −0.111; *p* = 0.401	r = −0.274; *p* = 0.036	r = −0.357; *p* = 0.005
WiPI	r = −0.160; *p* = 0.225	r = −0.177; *p* = 0.179	r = −0.319; *p* = 0.014	r = −0.391; *p* = 0.014
WoAUC	r = −0.174; *p* = 0.192	r = −0.197; *p* = 0.138	r = −0.321; *p* = 0.014	r = −0.403; *p* = 0.002
WiWoAUC	r = −0.160; *p* = 0.229	r = −0.214; *p* = 0.106	**r = −0.346**; ***p* = 0.008**	**r = −0.401**; ***p* = 0.002**
WoR	r = −0.123; *p* = 0.356	r = −0.155; *p* = 0.245	r = −0.306; *p* = 0.019	r = −0.355; *p* = 0.006
RT	r = 0.037; *p* = 0.782	r = −0.118; *p* = 0.374	r = −0.084; *p* = 0.528	r = −0.018; *p* = 0.893
mTTl	r = −0.160; *p* = 0.226	r = 0.024; *p* = 0.860	r = 0.045; *p* = 0.733	r = −0.015; *p* = 0.913
TTP	r = −0.007; *p* = 0.959	r = −0.216; *p* = 0.100	r = −0.178; *p* = 0.178	r = −0.092; *p* = 0.489
FT	r = 0.113; *p* = 0.398	r = −0.105; *p* = 0.433	r = −0.047; *p* = 0.724	r = −0.013; *p* = 0.923

The strongest correlations are indicated in bold. MeanLin—analyzed as a representative parameter of signal intensity—shows the strongest correlations as a representative parameter of signal intensity.

**Table 6:** Correlation of CEUS-based signal intensity parameters with preoperative split kidney function and postoperative kidney function in donors.

	Correlation with Split DTPA	Correlation with Split eGFR (CKD-EPI)	Correlation with Split eGFR (CG)	Correlation with Split Kidney Volume
Preoperative split kidney function				
MeanLin	r = −0.150; *p* = 0.253	r = −0.176; *p* = 0.179	**r = −0.331**; ***p* = 0.010**	**r = −0.398**; ***p* = 0.002**
WiWoAUC	r = −0.151; *p* = 0.258	r = −0.216; *p* = 0.104	**r = −0.338**; ***p* = 0.009**	**r = −0.389**; ***p* = 0.003**
Postoperative kidney function				
MeanLin	n.A.	r = −0.133; *p* = 0.483	**r = −0.399;** ***p* = 0.029**	n.A.
WiWoAUC	n.A.	r = −0.148; *p* = 0.436	**r = −0.393;** ***p* = 0.032**	n.A.

The strongest correlations are indicated in bold. n.A.: not available.
